# Novel Harmful Recessive Haplotypes Identified for Fertility Traits in Nordic Holstein Cattle

**DOI:** 10.1371/journal.pone.0082909

**Published:** 2013-12-20

**Authors:** Goutam Sahana, Ulrik Sander Nielsen, Gert Pedersen Aamand, Mogens Sandø Lund, Bernt Guldbrandtsen

**Affiliations:** 1 Center for Quantitative Genetics and Genomics, Department of Molecular Biology and Genetics, Aarhus University, Tjele, Denmark; 2 Knowledge Center for Agriculture, Aarhus, Denmark; 3 Nordic Cattle Genetic Evaluation, Aarhus, Denmark; Wageningen UR Livestock Research, The Netherlands

## Abstract

Using genomic data, lethal recessives may be discovered from haplotypes that are common in the population but never occur in the homozygote state in live animals. This approach only requires genotype data from phenotypically normal (i.e. live) individuals and not from the affected embryos that die. A total of 7,937 Nordic Holstein animals were genotyped with BovineSNP50 BeadChip and haplotypes including 25 consecutive markers were constructed and tested for absence of homozygotes states. We have identified 17 homozygote deficient haplotypes which could be loosely clustered into eight genomic regions harboring possible recessive lethal alleles. Effects of the identified haplotypes were estimated on two fertility traits: non-return rates and calving interval. Out of the eight identified genomic regions, six regions were confirmed as having an effect on fertility. The information can be used to avoid carrier-by-carrier mattings in practical animal breeding. Further, identification of causative genes/polymorphisms responsible for lethal effects will lead to accurate testing of the individuals carrying a lethal allele.

## Introduction

Alleles that cause an organism to die only when present in homozygous state are called recessively lethal alleles. Among individuals alive, such alleles only occur in heterozygotes. Such complete recessively lethal allele does not affect a carrier's reproductive performance. It may be passed down through many generations before meeting another copy. At low frequencies their evolution is essentially governed by genetic drift.

Genotyping technology now enables population researchers to genotype thousands of SNPs to cover the whole genome. With this genomic data, lethal recessives may be discovered from haplotypes that are common in the population but never occur in the homozygote state in live animals [1]. This approach only requires genotype data on phenotypically normal (i.e. live) individuals and not on the affected embryos that die. Following this approach VanRaden et al. [1] discovered 5 recessive lethal haplotypes in 3 cattle breeds. Fritz et al. [2] used the same approach in three French cattle breeds and identified a number of genomic regions showing a deficit in homozygotes. In the present study we followed the same approach to examine whether the three recessive haplotypes identified in the North American Holstein population segregate in the Nordic Holstein population and also to scan for the presence of additional recessive haplotypes in the Nordic Holsteins.

The objectives of the present study were to (1) test if the recessive lethal haplotypes reported by VanRaden et al. [1] were segregating in Nordic Holstein cattle population, (2) scan the whole genome to identify additional lethal haplotypes, (3) estimate the effected of identified haplotypes on female fertility.

## Materials and Methods

DNA was extracted from semen samples from bulls for genotyping in a previous project [3], so no ethical approval was required for this study.

### Genotyping of animals

A total of 7,937 Nordic Holstein bulls were genotyped with the BovineSNP50 BeadChip (Illumina, San Diego, CA, USA). Only SNPs with minor allele frequency of at least 0.05 and an average GenCall score (an indicator of the reliability of the genotypes called) of at least 0.65 were retained for analysis. A total of 36,387 SNPs on 29 bovine autosomes (BTAs) were selected for analyses. Individual SNP typings with GenCall less than 0.6 were dropped. The number of SNPs included for analysis varied from 675 on BTA28 to 2,320 on BTA1. The details on the genotyping platform and quality control for SNPs are described by Sahana et al. [3] The SNP positions within a chromosome were based on the *Bos taurus* genome UMD3.1 assembly [4]. Imputation of sporadically missing markers and phase determination was performed using Beagle software v. 3.3 [5]. Beagle was run with the default scale and shift parameters; no information on the relationships between individuals was used, and the outputs used for further analysis were the most probable genotypes.

Two sizes of marker windows were used to construct haplotypes to test: Either 25 or 75 consecutive markers were tested as haplotypes. All the ‘recessively lethal haplotypes’ identified by the haplotype length 75 contained one or more identified 25-SNP haplotype (Table S1). Therefore, only the results for haplotypes of length 25 are presented here. The haplotypes were labeled by chromosome number followed by the marker order of the left-most SNP of the haplotype.

The expected number of homozygous individuals for any particular haplotype was calculated using the number of genotyped animals divided by 4 and multiplied by the square of the carrier frequency assuming random mating. The expected number of homozygotes estimated this way may be overestimated if inbreeding was avoided or underestimated if allele frequencies changed over time [1]. The average inbreeding coefficient in Nordic Holsteins population used in this study were 3.7±1.9% and was close to the estimate reported by VanRaden et al. [6] from American Holstein population (5.5±1.9%).

To qualify as a candidate recessive lethal haplotypes, the criteria were first that based on the haplotype frequency the minimum expected number of homozygotes was six, and second that none was observed. When six homozygotes are expected assuming a Poison distribution corresponds to probability of 0.0025 for observing no homozygotes if the haplotype is not lethal. For confirmation of haplotypes identified by VanRaden et al. [1], we set the criteria that the number of expected homozygotes was at least 6 while we observed only one or none. The relaxed criterion for confirmation was kept in view of errors in imputation of genotypes and/or haplotype phase reconstruction and to allow for incomplete LD between the lethal allele the marker haplotype.

When more than one homozygote deficient haplotype overlapped, we estimated the correlation of carrier status on the basis of whether the bull is heterozygote ( = 1) or not ( = 0). Strong correlation between the carrier statuses of consecutive haplotypes indicates that the observed loss of homozygotes may be due to the same causative polymorphism.

### Fertility traits analyzed

The effects of the recessive lethal carrier-mating on non-return rate (NR) and calving interval (CI) were investigated using insemination records from matings involving carrier-sires with dam having carrier-sire. The NR is defined as the cow that is not subsequently rebred (not inseminated) within a specified period of time after an insemination. NR is recorded as binary measure of whether a cow returns to heat within a given period after the first insemination. In general, the longer the time interval, the smaller is the NR due to embryo loss and additional opportunity to observe heat in non-pregnant cows. NR at a shorter interval (e.g. 56 days) provide earlier information for genetic evaluation of bulls, but longer intervals (e.g. 150 days) generally give more accurate information on embryo loss. Therefore we studied non-return rates in cows at three different time-points after first insemination. The other trait analyzed was calving interval. The average calving interval is expected to be longer in case of carrier-by-carrier mating as they may result in embryo loss. A total of four traits were analyzed.

1. 0–56 days non-return rate per first insemination (NR56)
2. 0–100 days non-return rate per first insemination (NR100)
3. 0–150 days non-return rate per first insemination (NR150)
4. Calving interval in days (CI)

Data about inseminations and calvings was extracted from the Danish Cattle database covers back to 1984 (http://www.nordicebv.info). Information from lactation 1–3 was used. Only cows with known carrier sire were selected. By doing the analyses conditional on the parents'/grand parents' carrier status fertility effects in carrier cows other than the homozygous lethality in the embryo will not be important.

### Statistical analyses

We grouped daughters by whether their sire was a carrier or not. The analysis was restricted to only those mating involving carrier-sires and the females with carrier-sire (i.e. maternal grandsire (mgs) of the embryo). In such cases the probability of the female being carrier is 0.5 and the embryo receiving a copy of the recessive lethal polymorphism from its carrier mgs is 0.25. As we are looking for effect of a recessive allele it will only be observed in case of carrier-carrier mating.

The probability of a fetus being homozygous (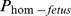) for the recessively lethal allele carrying haplotype, was estimated from the probability of getting recessive haplotype from both the sire (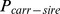) and the maternal grandsire (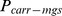) of the fetus.
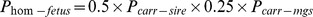



The variation of each fertility trait was analyzed by means of covariance, where the regression coefficient on 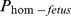
was the parameter of interest.

The effect was analyzed by the following model:
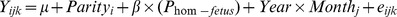



Where *Y_ijk_* is the fertility traits (NR and CI) recorded on females; μ is the adjusted mean; *Parity_i_* is the effect of i^th^ parity (*i* = 1, 2, 3); β is the regression coefficient of the fertility trait on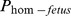; Y*ear × Month_j_* is the insemination season within year for the insemination calving, and *e_ijk_* is the random residual. The effects were estimated by SAS by separate analysis for each fertility trait.

## Results

The means, standard deviations and phenotypic correlations of the four fertility traits analyzed in this study are given Table 1.

**Table 1:** Mean, standard deviation (Std. Dev.) and phenotypic correlation for the analyzed fertility traits.

Trait	No. of records	Mean	Std. Dev	Phenotypic correlations			
				NR56	NR100	NR150	CI
**NR56**	513,566	59.41	49.00	1.00			
**NR100**	515,332	48.58	49.98	0.80	1.00		
**NR150**	515,364	45.67	49.80	0.76	0.94	1.00	
**CI**	396,173	405.04	71.61	−0.21	−0.38	−0.46	1.00

NR – Non-return rate, CI – Calving interval.

### Possible recessive lethal haplotypes

A total of 17 haplotypes on six chromosomes with absence/deficiency of homozygotes were identified (Table 2). These 17 haplotypes could be loosely grouped into eight genomic regions based on their relative proximity. The probability of not observing any homozygotes ranged from 0.002 with 6 expected homozygotes on BTA11 to 2.32×10^−16^ with 36 expected homozygotes on BTA7. The carrier frequencies of these haplotypes ranged from 2.7 to 6.7%.

**Table 2:** Locations and carrier frequencies for potential lethal haplotypes in Nordic Holstein population.

Hap ID	Start position	Start marker	End position	End marker	Carrier frequency	No. of homozygotes	
						Obs	Exp
**05–826**	66830677	Hapmap59828-rs29027014	68632534	ARS-BFGL-NGS-33119	0.033576	1	9
**05–1351**	106713645	ARS-BFGL-NGS-44754	107590490	ARS-BFGL-NGS-16163	0.040317	1	13
**05–1476**	112647134	BTA-75143-no-rs	114405063	Hapmap41631-BTA-75177	0.032065	1	8
**07–126**	6608007	BFGL-NGS-118326	10907840	BTB-01568825	0.067594	0	36
**07-501**	34633456	BTB-01947935	36127497	BTB-01172317	0.038427	0	12
**08–1276**	83888935	BTA-14515-no-rs	85392078	Hapmap41653-BTA-82121	0.029734	0	7
**08–1301**	85482389	Hapmap39282-BTA-63744	86718128	BTB-00301689	0.029986	0	7
**08–1326**	86780525	Hapmap47937-BTA-39125	88464533	Hapmap49329-BTA-82142	0.030364	0	7
**08–1351**	88545459	BTB-00364735	89859523	Hapmap61061-rs29020453	0.030679	0	7
**11–926**	55345639	Hapmap38708-BTA-88091	57276682	Hapmap60754-rs29010392	0.027529	0	6
**11–976**	59186199	Hapmap60204-rs29026371	60572203	BTA-99085-no-rs	0.027403	0	6
**11–1001**	60602028	BTA-99088-no-rs	62144546	Hapmap40862-BTA-100125	0.027214	0	6
**11–1026**	62211117	Hapmap34935-BES11_ Contig422_2192	63759322	ARS-BFGL-NGS-41687	0.026899	0	6
**19–151**	13154786	ARS-BFGL-NGS-37177	14478389	ARS-BFGL-NGS-62115	0.038049	0	11
**21–276**	20477690	ARS-BFGL-NGS-24595	21730828	ARS-BFGL-NGS-2185	0.040128	0	13
**21–301**	21796083	ARS-BFGL-NGS-73451	22858538	BFGL-NGS-112524	0.041136	1	13
**21–326**	22898102	ARS-BFGL-NGS-11578	24844501	Hapmap51463-BTA-51879	0.038868	1	12

The SNP loci were based on *Bos taurus* genome assembly (UMD 3.1); Obs – Observed; Exp – Expected.

On BTA5, we observed three haplotypes at 66.8, 106.7 and 112.6 Mb with a deficiency, but not a total absence of homozygotes (Table 3).The haplotype at 66.8 Mb (05–826) did not show any significant effect on any of the four fertility traits analyzed. The two other haplotypes (05–1351 and 05–1476) increased the calving intervals significantly by 16.8 and 22.2 days respectively. However, none of them showed any significant effect on the non-return rates.

**Table 3:** Regression coefficient for prediction of different fertility traits from the probability of the fetus being homozygous for the potential recessive lethal haplotype (*P_hom-fetus_*).

Hap-ID	Effect of 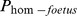 on							
	NR56		NR100		NR150		Calving interval	
	Effect	p-value	Effect	p-value	Effect	p-value	Effect	p-value
**05–826**	−4.5	0.45^NS^	−5.3	0.38^NS^	−5.68	0.35^NS^	15.4	0.11^NS^
**05–1351**	−6.0	0.19^NS^	−2.4	0.60^NS^	−3.26	0.48^NS^	16.8	0.02
**05–1476**	−7.2	0.13^NS^	−5.2	0.29^NS^	−5.3	0.28^NS^	22.2	0.003
**07–126**	0.1	0.72^NS^	1.3	0.54^NS^	1.6	0.46^NS^	3.6	0.12^NS^
**07–501**	−6.1	0.0001	−6.7	0.0001	−6.2	0.0001	2.4	0.44^NS^
**08–1276**	−15.3	0.05	−11.9	0.13^NS^	−13.0	0.10^NS^	28.4	0.02
**08–1301**	−20.8	0.003	−20.1	0.004	−20.4	0.004	22.8	0.04
**08–1326**	−21.4	0.002	−21.3	0.002	−21.5	0.002	22.7	0.04
**08–1351**	−18.3	0.008	−18.1	0.001	−18.1	0.001	27.2	0.01
**11–926**	−14.1	0.0008	−16.5	0.0001	−17.1	0.0001	15.4	0.02
**11–976**	−13.7	0.001	−16.9	0.0001	−17.5	0.0001	15.4	0.02
**11–1001**	−13.5	0.001	−16.4	0.0001	−17.1	0.0001	15.7	0.02
**11–1026**	−14.7	0.001	−16.7	0.001	−17.0	0.0001	13.1	0.02
**19–151**	−14.4	0.0001	−11.1	0.01	−10.6	0.02	6.7	0.34^NS^
**21–276**	−9.7	0.01	−24.4	0.0001	−25.0	0.0001	39.7	0.0001
**21–301**	−9.0	0.01	−23.6	0.0001	−25.7	0.0001	43.1	0.0001
**21–326**	−7.2	0.06	−22.3	0.0001	−25.0	0.0001	47.2	0.0001

NR – Non-return rate; ^NS^ – not significant (p>0.05).

Two haplotypes on BTA7, 07–126 (6.6 Mb) and 07–501 (34.6 Mb) had carrier frequencies of 6.8% and 3.8% respectively, but no homozygous individuals were observed (Table 3). The haplotype 07–126 had the highest carrier frequency (6.7%) among all the 17 haplotypes identified but no homozygote individuals were observed. It did not show any significant effect on any of the four fertility traits analyzed in this study. The haplotype (07–501) showed highly significant effects (p<0.0001) on all the three non-return rates. There was a decrease of about 6% in NR comparing mattings with a (hypothetical) probability of a fetus being homozygous (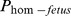) was 1 compared to a mating with 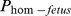 = 0.

On BTA8, there were four consecutive haplotypes between 83.88–89.85 Mb each with 7 homozygotes expected but none were observed. Out of these four haplotypes, 08–1301 (85.48 Mb), 08–1326 (86.78 Mb) and 08–1351 (88.54 Mb) significantly (p≤0.05) affected all the four fertility traits analyzed (Table 3), while 08–1276 (83.8 Mb) showed a significant (p≤0.05) effect on NR56 and CI. These haplotypes lowered the non-return rate by 15.3 to 21.5% and increased the calving intervals by 22.7 to 27.2 days. The correlation among the carrier status of the bulls (1 for carrier and 0 for non-carrier) among these four haplotypes varied between 0.85 and 0.97.

On BTA11 four closely spaced haplotypes between 55.34 and 63.75 Mb exhibited a complete absence of homozygotes. For all these four haplotypes, the numbers of homozygotes were expected to be six (carrier frequency  = 2.7%) while none were observed. All four haplotypes had a significant effect (P≤0.02) on all the four fertility traits analyzed (Table 3). In carrier-by-carrier mattings NR was lowered by 13.5 to 18.1%, while there was 13 to 15 days increase in CI. The correlation among the sires' carrier status for these closely located haplotypes were between 0.80 and 0.98.

One haplotype on BTA19 at 13.15 Mb (19–151) had an expected number of homozygotes of 11 with none observed. This haplotype had a significant effect (p≤0.02) on the three non-return rates analyzed but not on calving interval (Table 3). The regression coefficients of 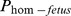
on non-return rates varied between −10.6% for NRR150 to −14.4% for NRR56.

We observed 3 consecutive haplotypes (21–276, 21–301 and 21–326) between 20.47–24.84 Mb on chromosome 21, where the expected numbers of homozygotes were between 12 and 13 while one or none were observed. All these three haplotypes had significant effects on all the four fertility traits analyzed in this study. The locations of these haplotypes overlap with the location of the Brachyspina locus [7], [8]. The correlation among the sires' carrier status for these three haplotypes varied between 0.95 and 0.96. The regression coefficients of 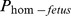
on NR varied between −7.2 to −25.7. The effects of 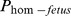
on the CI were between 39.7 and 47.2 days (Table 3).

## Discussion

Illumina 50 k genotyping data from the Nordic Holstein population were analyzed to identify possible recessively lethal haplotypes. We examined whether the fertility performance was significantly different between the carrier-by-carrier mattings and other mattings for the identified haplotypes. This was done fitting the probability of a fetus being homozygous for a putative recessively lethal allele based on the carrier status of the sire and the dam. The effects were estimated on four fertility traits, three of them were non-return rates at different lengths and the fourth fertility trait was calving interval.

VanRaden et al. [1] identified three recessive lethal haplotypes in North American Holstein population on chromosome 1, 5 and 8. In the present study we observed a haplotype (05–826) at 66.8–68.6 Mb on BTA5, which was deficient in homozygotes, but does not overlap with the location of HH1 of VanRaden et al. (58–66 Mb).This haplotype showed significant effects on NR56 and NR100, but not on CI. Two new haplotypes located at 106.7–114.4 on BTA5 were observed to have a deficiency of homozygotes, but not a total absence of homozygotes. Both the haplotypes increased the calving intervals significantly, and though they also lowered the NR but the estimates were not significant. The correlation between the sires' carrier status for these two haplotypes was 0.93, which means the effect observed for both the haplotypes may be due to a single polymorphism located in this genomic region.

Another possible recessively lethal haplotype, HH3, on BTA8 (90–95 Mb) reported by VanRaden et al. [1] was confirmed in the Nordic Holstein population between 83.4–89.9 Mb. There were four consecutive haplotypes with no homozygotes observed. The 3 haplotypes were close to the HH3 location. They had significant effects on all the four fertility traits.

We observed three consecutive 25-SNP haplotypes between 20.4–24.8 Mb on BTA21. All these three haplotypes showed significant effects both on non-return rates and calving interval. The locations of these haplotypes are close to or overlap with location of the known defect at the Brachyspina locus on BTA21 [7], [8]. A 3.3 Kb deletion encompassing exons 25 to 27 of the bovine gene FANCI (21.13–21.19 Mb on BTA21) causes the Brachyspina phenotype when homozygous [8]. The haplotypes identified in this study on BTA21 in Nordic Holsteins are therefore due to the Brachyspina syndrome and the haplotype 21–276 carries the disease gene. Charlier et al. [8] reported a 7.4% carrier frequency of this recessive defect in the Dutch Holstein-Friesian breed while we observed the carrier frequency of 4% in the Nordic Holstein population.

VanRaden et al. [1] identified a recessive lethal haplotype (BH1 on BTA7 at 41–47 Mb) in the Brown Swiss population. We observed a haplotype with a highly deficiency of homozygotes at 6.61 Mb on BTA7, but with no significant effect on either non-return rates or calving interval. We identified a haplotype at 33.3–34.6 Mb, which was affecting non-return rate in Nordic Holsteins. It is highly unlikely that this identified haplotype is the same as the BH1 due to difference in the genomic locations and also from a different cattle breed.

We observed two additional haplotypes on BTA11 and BTA19 with no homozygotes observed. While all the four consecutively located haplotypes on BTA11 were affecting both NR and CI, the lone haplotype on BTA19 was only affecting NR. The high correlation of carrier status of the four haplotypes on BTA11 indicated that all these four haplotypes were representing the same causative polymorphism responsible for the lethal effect.

## Conclusions

Absence of homozygotes can be used to reveal carrier haplotypes for new defects. We have identified eight genomic regions harboring possible recessively lethal alleles. Six of them were confirmed having effects on fertility related traits including one previously reported (Brachyspina) and five novel recessively lethal loci. The information can be used to avoid carrier-by-carrier mattings and select against the underlying recessive lethals in practical cattle breeding. Further, identification of causative polymorphism responsible for the lethal effect will lead to accurate testing of the individuals carrying the lethal allele.

## Supporting Information

Table S1 Haplotypes with 75 consecutive makers generally contains the ‘25-marker’ haplotype (in red) with missing homozygotes.
(DOCX)

## References

[1] VanRaden PM, Olson KM, Null DJ, Hutchison JL (2011) Harmful recessive effects on fertility detected by absence of homozygous haplotypes. J Dairy Sci 94: 6153–6161. 22118103 10.3168/jds.2011-4624

[2] Fritz S, Capitan A, Djari A, Rodriguez SC, Barbat A, et al (2013) Detection of Haplotypes Associated with Prenatal Death in Dairy Cattle and Identification of Deleterious Mutations in GART, SHBG and SLC37A2. PLOS ONE 8(6): e65550. 23762392 10.1371/journal.pone.0065550 PMC3676330

[3] Sahana G, Guldbrandtsen B, Bendixen C, Lund MS (2010) Genome-wide association mapping for female fertility traits in Danish and Swedish Holstein cattle. Anim Genet 41: 579–588. 20477799 10.1111/j.1365-2052.2010.02064.x

[4] Zimin AV, Delcher AL, Florea L, Kelley DR, Schatz MC, et al (2009) A whole-genome assembly of the domestic cow, Bos taurus. Genome Biol 10: R42. 19393038 10.1186/gb-2009-10-4-r42 PMC2688933

[5] Browning BL, Browning SR (2009) A unified approach to genotype imputation and haplotype phase inference for large data sets of trios and unrelated individuals. Am J Hum Genet 84: 210–223. 19200528 10.1016/j.ajhg.2009.01.005 PMC2668004

[6] VanRaden PM, Olson KM, Wiggans GR, Cole JB, Tooker ME (2011) Genomic inbreeding and relationships among Holsteins, Jerseys, and Brown Swiss. J Dairy Sci 94: 5673–5682. 22032391 10.3168/jds.2011-4500

[7] Agerholm JS, McEvoy F, Arnbjerg J (2006) Brachyspina syndrome in a Holstein calf. J Vet Diagn Invest 18: 418–422. 16921889 10.1177/104063870601800421

[8] Charlier C, Agerholm JS, Coppieters W, Karlskov-Mortensen P, Li W, et al (2012) A Deletion in the Bovine FANCI Gene Compromises Fertility by Causing Fetal Death and Brachyspina. PLOS ONE 7(8): e43085. 22952632 10.1371/journal.pone.0043085 PMC3430679

