# Supplementary material for: Novel Harmful Recessive Haplotypes Identified for Fertility Traits in Nordic Holstein Cattle
Source: PLoS One. 2013 Dec 20;8(12):e82909. doi: 10.1371/journal.pone.0082909 (PMC3869739; doi:10.1371/journal.pone.0082909)
Supplement: Table S1 — Haplotypes with 75 consecutive makers generally contains the ‘25-marker’ haplotype (in red) with missing homozygotes. (DOCX) [file pone.0082909.s001.docx]

**Table S1 Haplotypes with 75 consecutive makers generally contains the ’25-marker’ haplotype (in red) with missing homozygotes.**

| Chr. | Start Bp | End Bp | haplotype | Frequency |
| --- | --- | --- | --- | --- |
| 7 | 10254576 | 11248845 | 111112313322112113133313113133221213323133111123112123223131231212133133311 | 0.022 |
| 7 | 82154652 | 82376045 | 333213331331331113111331311111311221333113222333133321323113132133333323111 | 0.021 |
| 11 | 13967660 | 14344322 | 131313113311113111133122112311113111331113113111113333313331333312331311113 | 0.022 |
| 11 | 58853668 | 59093179 | 111133131133111311323313333123132333311213121131133231333313133111313133111 | 0.022 |
| 11 | 59566123 | 60082013 | 133313133131313113113313323133131323123133311331331311312113313113221332321 | 0.022 |
| 11 | 60114494 | 60401670 | 333132113313313311211111123133123111111333313311113311111311313133111133111 | 0.021 |
| 21 | 20351451 | 20584082 | 133313111323123113331321333111331133113113313231312333311313113131331311131 | 0.040 |
| 21 | 21078976 | 21340975 | 121312133313131133331311113123111113111111131311131221331121131111111111321 | 0.036 |
| 21 | 21344766 | 21602108 | 133113333133331131321131112311333333133211211331123333311121131131132111131 | 0.035 |
